# The Effects of a Mediterranean Diet Intervention on Targeted Plasma Metabolic Biomarkers among US Firefighters: A Pilot Cluster-Randomized Trial

**DOI:** 10.3390/nu12123610

**Published:** 2020-11-24

**Authors:** Mercedes Sotos-Prieto, Miguel Ruiz-Canela, Yiqing Song, Costas Christophi, Steven Mofatt, Fernando Rodriguez-Artalejo, Stefanos N. Kales

**Affiliations:** 1 Department of Preventive Medicine and Public Health, School of Medicine, Universidad Autónoma de Madrid, IdiPaz (Instituto de Investigación Sanitaria Hospital Universitario La Paz), Calle del Arzobispo Morcillo 4, 28029 Madrid, Spain; fernando.artalejo@uam.es; 2 Biomedical Research Network Centre of Epidemiology and Public Health (CIBERESP), Carlos III Health Institute, 28029 Madrid, Spain; 3 Department of Environmental Health, Harvard T.H. Chan School of Public Health, Boston, MA 02115, USA; costas.christophi@cut.ac.cy (C.C.); stefokali@aol.com (S.N.K.); 4 Department of Preventive Medicine and Public Health, IdiSNA, University of Navarra, 31009 Pamplona, Spain; mcanela@unav.es; 5 Biomedical Research Network Centre for Pathophysiology of Obesity and Nutrition (CIBEROBN), Carlos III Health Institute, 28029 Madrid, Spain; 6 Department of Epidemiology, Richard M. Fairbanks School of Public Health, Indiana University, Indianapolis, IN 46202, USA; yiqsong@iu.edu; 7 Cyprus International Institute for Environmental and Public Health, Cyprus University of Technology, 30 Archbishop Kyprianou Str., 3036 Lemesos, Cyprus; 8 National Institute for Public Safety Health, Indianapolis, IN 46204, USA; steven.mofatt@ascension.org; 9 IMDEA-Food Institute, CEI UAM+CSIC, 28049 Madrid, Spain; 10 Department of Occupational Medicine, Cambridge Health Alliance, Harvard Medical School, Cambridge, MA 02145, USA

**Keywords:** Mediterranean Diet, metabolites, clinical trial, lipoprotein composition, biomarkers

## Abstract

Metabolomics is improving the understanding of the mechanisms of the health effects of diet. Previous research has identified several metabolites associated with the Mediterranean Diet (MedDiet), but knowledge about longitudinal changes in metabolic biomarkers after a MedDiet intervention is scarce. A subsample of 48 firefighters from a cluster-randomized trial at Indianapolis fire stations was randomly selected for the metabolomics study at 12 months of follow up (time point 1), where Group 1 (*n* = 24) continued for another 6 months in a self-sustained MedDiet intervention, and Group 2 (*n* = 24), the control group at that time, started with an active MedDiet intervention for 6 months (time point 2). A total of 225 metabolites were assessed at the two time points by using a targeted NMR platform. The MedDiet score improved slightly but changes were non-significant (intervention: 24.2 vs. 26.0 points and control group: 26.1 vs. 26.5 points). The MedDiet intervention led to favorable changes in biomarkers related to lipid metabolism, including lower LDL-C, ApoB/ApoA1 ratio, remnant cholesterol, M-VLDL-CE; and higher HDL-C, and better lipoprotein composition. This MedDiet intervention induces only modest changes in adherence to the MedDiet and consequently in metabolic biomarkers. Further research should confirm these results based on larger study samples in workplace interventions with powerful study designs.

## 1. Introduction

Currently, the understanding of diet-health relationships has gradually shifted from individual dietary components to overall dietary patterns that beneficially modulate metabolic physiology [1]. In this regard, several epidemiological and clinical studies have shown that the traditional Mediterranean-style eating pattern (characterized by high intake of fruits and vegetables, olive oil, legumes, whole grains, and fish and moderate consumption of white meat, dairy and wine during meals) has many health benefits [2,3], including beneficial changes in biomarkers of CVD risk [4,5] and lower risk of cardiovascular disease (CVD) [6,7,8].

However, the exact mechanisms of the benefits of the Mediterranean-style dietary pattern have yet to be understood. The application of metabolomics to measure changes in biological variables in response to dietary interventions has been proposed as a potential tool to discover biomarkers associated with healthier eating [9,10,11,12,13]. There is observational epidemiological evidence that acylcarnitines, Trimethylamine N-oxide (TMAO), some amino acids such as phenylalanine, glutamate as well as several lipid classes are associated with CVD risk [14]. While the effects of individual dietary ingredients on metabolome have been reported [15,16,17,18,19,20], only a few studies have focused on overall dietary patterns [21,22,23,24,25,26]. Some cross-sectional studies such as the Whitehall II study showed that a healthy diet was associated with specific fatty acids that reduced the risk of CVD [27]. In a British population, the association between the adherence to the MedDiet and cardiometabolic outcomes was mediated by baseline levels of acylcarnitines, sphingolipids, and phospholipids [24]. In the Supplementation en Vitamines et Mineraux Antioxydants (SU.VI.MAX) study, some metabolites were also cross-sectionally associated with dietary recommendations [28]. More recently, a metabolic signature of the Mediterranean Diet has been consistently identified in two large cohorts [26].

Importantly, studies analyzing changes in metabolites levels in response to MedDiet interventions are still scarce and inconclusive. In the Metabolic Syndrome Reduction in Navarra (RESMENA)study, after 2 months of an energy restricted MedDiet intervention, some significant changes in metabolites were shown but they were no longer observed after 6 months [22]. Additional evidence comes from the Prevention with Mediterranean Diet (PREDIMED) study, where several a priori-designed analyses found that the MedDiet may reduce the deleterious effect on cardiovascular or type-2 diabetes risk associated with 1 year changes in branched-chain amino acids [29,30], carnitines [31] and other metabolites. However, in most of these analyses, the 1 year metabolite changes were similar between the intervention and the control group, suggesting that the observed protective effect of the MedDiet could be due to other mechanisms.

Therefore, the objective of this study is to identify changes in plasma metabolic biomarkers associated with a MedDiet intervention within a subsample of a cluster-randomized controlled trial (Feeding America’s Bravest) among firefighters. We hypothesize that the MedDiet intervention induces changes in metabolites within clinically relevant pathways.

## 2. Materials and Methods

### 2.1. Study Design and Participants

**Figure 1:**
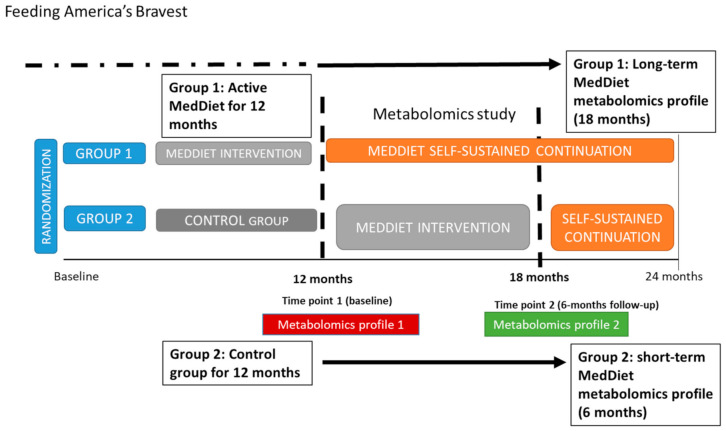
Study design and timeline of the metabolomics study within Feeding America´s Bravest parent study (step-wedge cluster-randomized control trial).

The overall study design, intervention strategies and primary outcomes of the Feeding America’s Bravest trial have been previously reported [32]. Briefly, Feeding America’s Bravest is a step-wedge cluster-randomized controlled trial within the 44 stations of Indianapolis Fire Department, which aims to compare a MedDiet Nutritional Intervention vs. an ad libitum Midwestern-style diet (control or no intervention) during 12 months. Group 1 (or the intervention group for 12 months) continued under a self-sustained continuation phase for another 12 months. Group 2 (initially controls) crossed over (at 12 months) to receive the active Mediterranean Diet Nutritional Intervention for 6 months followed by another 6 months of a self-sustained intervention phase. For this nested study, we randomly selected a sub-group of participants (*n* = 48) whose fire stations had been assigned to the MedDiet intervention (*n* = 24) or the control group (*n* = 24) for the previous 12 months. At that time (time point 1 for our study and 12 months follow up of the parent study), the intervention group (Group 1) continued under a self-sustained phase for another 6 months (time point 2 for our study or 18 months of the parent study) and the control group underwent the active MedDiet intervention for 6 months. Plasma metabolic biomarkers were analyzed at the two time points (Figure 1). The overarching Feeding America’s Bravest protocol was approved by the Harvard Institutional Review Board (IRB16-0170) and is registered at Clinical Trials (NCT02941757). All participants provided informed consent for participation.

### 2.2. Mediterranean Diet Intervention

At the two time points of this study, data on sociodemographic characteristics, physical activity, sleep behaviors (a modified Pittsburgh Sleep Quality Index, where on a scale from 0 to 7 participants were asked to identify the statement which option best described their habitual level of physical activity over the past month [33]), food consumption based on a self- reported validated food frequency questionnaire [34] and a modified Mediterranean Diet scale [35], and anthropometric and clinical variables were assessed.

The MedDiet intervention has been described in detail elsewhere [32]. Briefly, it consisted of educational sessions and videos created by a certified nutritionist, leaflet and recommendations about the Mediterranean Dietand lifestyle, firefighter-tailored Mediterranean recipes (by modifying Firefighters´ favorite recipes according to the MedDiet principles by a chef and a nutritionist), in-site chef cooking demonstration, a firefighters’ food pyramid, Mediterranean food samples and discount coupons to a large supermarket chain for specific Mediterranean Diet-compatible foods [32].

Adherence to the MedDiet was assessed by the validated modified Mediterranean Diet Score (mMDS) [35,36] and the PREDIMED score. (36) The mMDS range from 0 (lowest) to 51 (highest conformity to the MedDiet) [36] and consists of 13 domains including consumption of fast food, fruits, vegetables, legumes, nuts, sweet desserts, fried foods, ocean fish, breads and starches (consumed at home and the fire station), the type and frequency of alcoholic beverages, non-alcoholic beverages (consumed at home and the fire station) and the type of cooking oil or fat (consumed at home and the fire station). Each component ranged from 0 (less adherence) to 4 points (better adherence) except the type of alcohol (wine; 0–2 points) and the type of cooking oil or fat (0–5 points). Weighted scores was considered for domains evaluated at both the homes and fire stations based on the percentage of meals consumed at each location [36].

The PREDIMED score [37] consists of 14 questions; 12 of them are about food consumption frequency (olive oil consumption, vegetables, fruits, red/processed meats, butter/margarine, soda drinks, wine, legumes, fish/seafood, nuts, commercial sweets, sofrito consumption), and another two about food intake habits considered characteristic of the Spanish Mediterranean Diet (preference of poultry consumption instead of red meats, use of olive oil as main culinary fat). Each question was scored 0 or 1, with a total possible range of 0 to 14; higher scores indicate greater adherence to the MedDiet.

### 2.3. Collection of Plasma Samples

In this nested study, 12 h fasting blood samples were collected at time point 1 (baseline for this metabolomics study and 12 months for the trial) and time point 2 (6 months follow up for this metabolomics study and 18 months for the trial) of follow up; samples were kept cold and immediately processed to separate the plasma with a refrigerated centrifuge. Next, the 200 µL cryovials were placed at −80 °C.

### 2.4. Plasma Biomarkers Measurements

Metabolites were quantified in plasma samples from 83 individuals that had optimal values using high-throughput proton Nuclear Magnetic Resonance (NMR) metabolomics (Nightingale Health Ltd., Helsinki, Finland). This method provides simultaneous quantification of routine lipids, lipoprotein subclass profiling with lipid concentrations within 14 subclasses, fatty acid composition, and various low-molecular metabolites including amino acids, ketone bodies and glycolysis-related metabolites. Details of the experimentation and applications of the NMR metabolomics platform have been described previously [38]. All measured metabolites fall in the range of detection; representative coefficients of variations (CVs) for the metabolic biomarkers were published previously [39,40].

### 2.5. Statistical Analysis

Differences in baseline characteristics between the two groups were examined by *t*-test ANOVA for continuous variables (means ± standard deviation [SD]) or Chi-square for categorical data (percentages). Statistical analyses of the metabolites were performed on log-transformed data that were scaled to SD units to facilitate comparisons across metabolites. The effect of the MedDiet intervention between point 1 and point 2 in this analysis (6 months) was assessed using a linear mixed-effects model adjusted for age and sex. As many of the metabolites are biologically correlated, applying a multiple testing correction using all the 225 biomarkers would be too strict. Thus, account for multiple testing, Bonferroni correction was applied with significance level defined as 0.05/21 = 0.002, with 21 being the number of principal components that explained 99% of the variation in the NMR data.

Cross-sectional linear associations between the adherence to the mMDS score and metabolite concentrations at both time points for both the MedDiet intervention and control groups were obtained using a linear regression model adjusted for age and sex. The analyses were performed using ggforestplot R package (version 0.0.2) and the linear mixed-effects model was fitted with the nlme R package (version 3.1.-144).

## 3. Results

### 3.1. Participant Characteristics

Characteristics of the 48 participants at time point 1, and changes after 6 months follow up (time point 2), are summarized in Table 1. Of the 48 participants at time point 1, 44 (92%) were followed up and provided dietary information and anthropometric measures at time point 2. Information for the plasma metabolites were available for *n* = 47 at baseline and *n* = 36 at the end of follow up (Supplementary Materials Figure S1). There were no statistically significant differences in the adherence to the mMDS or PREDIMED score at time 1 and after the 6 months follow up within and between groups. Similarly, no differences were seen for age or sex (Table 1).

### 3.2. The 6 Month Effect of the MedDiet Intervention

**Figure 2:**
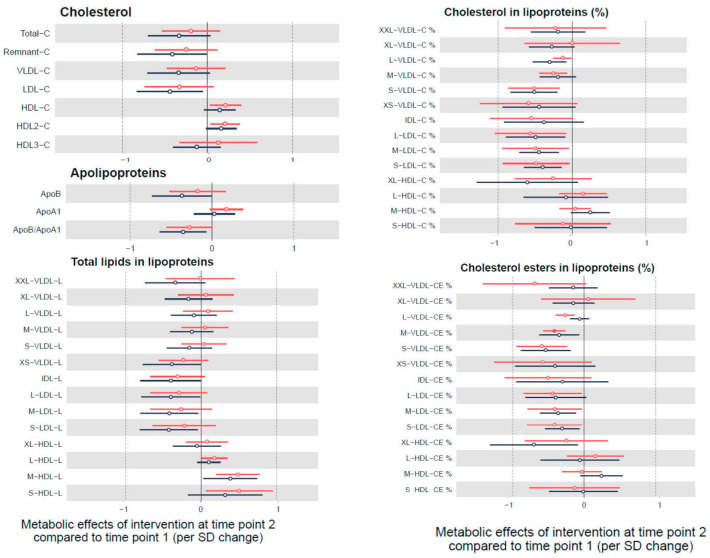
Metabolic effects of the intervention after 6 months follow up (time point 2) compared to baseline (time point 1) (linear mixed models adjusted by age and sex). Results show changes by SD in each metabolite per unit change in mMDS score and are displayed by hollow points. Only those significant results (after correction of multiple testing) are indicated by filled points along with their 95% confidence intervals. In black is shown Group 1 (the intervention group for 12 months) that continued under a self-sustained continuation phase for another 6 months. In red is shown Group 2 (control) that received the active Mediterranean Diet Nutritional Intervention for 6 months.

Figure 2 shows the 6 months effects of the intervention in the most relevant metabolites pathways. Data on the effect of the intervention in all the metabolites studied by group are presented in Table S1. The main subgroups affected by the intervention were the lipids and lipoproteins. Specifically, we observed a reduction in LDL-C, ApoB/ApoA1 ratio, remnant cholesterol, and higher HDL-C, and other subfractions such as lower cholesterol in L-VLDL-C, S-VLDL-C, L-LDL-C, M-LDL-C, S-LDL-C lipoproteins, and the composition of the lipoproteins after 6 months of intervention. Of note, these associations did not reach statistical significance after correcting the *p*-values for multiple testing (except for a decrease in M-VLDL-CE and an increase in lactate).

### 3.3. Cross-Sectional Association between Mediterranean Diet Adherence and Biomarkers

**Figure 3:**
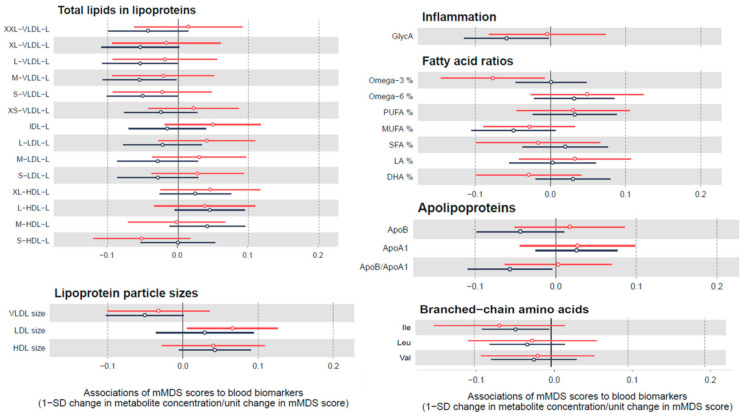
Cross-sectional association between biomarkers and adherence to the MedDiet for all participants at time point 1 and time point 2. Red lines show the results for the participants at baseline and black lines for the participants at follow up. Results show changes by SD in each biomarker per unit change in mMDS score and are displayed by hollow points along with their 95% confidence intervals.

We also examined the cross-sectional linear association between biomarkers and adherence to the MedDiet at point 1 and 2 of this study, regardless of the participant’s group at baseline and follow up. Results were similar at both time points, although somewhat higher effect sizes where observed at time 2 where all participants had received at least 6 months of the MedDiet intervention (Figure 3). A 1 unit difference in mMDS score was associated with lower total lipids in lipoproteins of different sizes (VLDL, LDL) and ApoB/ApoA1 ratio, lower concentrations of a marker of inflammation (Glyc A), lower concentrations of branched chain amino acids and higher polyunsaturated fatty acids (PUFAs) and Docosahexaenoic acid (DHA) (Figure 3). We further included all the participants grouped together and analyzed the association between unit changes in the MedDiet score and SD changes in metabolic markers. We found a similar pattern in the results, but not significant, with a tendency to higher lipoprotein particle size with higher MedDiet scores (Figure S2).

Similar results were found when we used the PREDIMED score instead of the mMDS (Figure S3).

## 4. Discussion

In this sub-study of firefighters in Indianapolis participating in a cluster-randomized MedDiet intervention trial, we found that the MedDiet intervention was associated with favorable changes in markers of cardiovascular risk, specifically those related to the lipid metabolism (cholesterol, lipid composition, or cholesterol esters in the VLDL, IDL, and LDL lipoprotein subclasses, and ApoB/ApoA ratio) that were non-significant after correcting for multiple testing (except for a decrease in M-VLDL-CE). When the adherence to the MedDiet was measured with a self-reported scale (mMDS), the direction of the association with metabolites was similar at both time points (baseline and 6 months after the follow up).

Our results highlighting the changes in plasma metabolites related to lipid metabolism are in line with other studies [20,26]. A recent investigation that identified a metabolic signature of adherence to the MedDiet showed that out of 67 metabolites, 45 were lipids followed by 19 amino acids, 2 vitamins and 1 xenobiotic [26]. Although we used a different methodology and a different set of biomarkers, and thus we could not replicate this metabolic signature, our results support that the MedDiet may induce changes in relevant lipid species and subclasses related to atherogenic risk. In fact, the MedDiet is high in healthy fats (>35–40% of the total energy) mostly from monounsaturated fatty acids (MUFAs) (olive oil mostly) and PUFAs (from nuts and fish), and therefore the results are not surprising. In the firefighter population, we previously reported good correlation between nutrient intake from the food frequency questionnaire and the corresponding plasma biomarkers (omega-3, Eicosapentaenoic acid (EPA), and DHA) [36]. In line with these results, we found that changes in the MedDiet scores showed some tendency to be associated with fatty acids (an increase in PUFA% specifically DHA% in the expense of MUFA%). Although olive oil is a main component of the MedDiet, previous research found that higher consumption of this oil was linked to changes in omega 3, but not MUFA concentrations [36,41,42]. In addition, the average olive oil consumption in the firefighters is only approximately 0.5 tbsp/day, which is similar to other US cohorts [43] but much lower than in a Spanish cohort (4 tbsp/day) [44]. Nonetheless, it looks like changes in omega 3 to fatty acid ratio, PUFA to FA ratio and DHA to FA ratio increase with changes in the adherence to the MedDiet. This is in line with other studies [20,36], and suggests that those biomarkers could serve as indicators of adherence to the MedDiet.

We found that the MedDiet intervention induced a decrease in total cholesterol, remnant-C, VLDL-C and LDL-C and an increase in HDL-C and HDL2-C. Many studies have already demonstrated the effect of the MedDiet on total lipid metabolism, especially reducing total cholesterol and increasing HDL-C [45]. For example, the PREDIMED study, a randomized control trial, found that those in the MedDiet intervention (with olive oil or nuts) over 6 months had an increase in HDL-C but not a reduction in LDL-C [45]. Other studies support that replacing dietary saturated fatty acids (SFAs) with PUFA reduces the plasma LDL-C and subsequently the risk of cardiovascular disease [46,47,48].

In our study, we also observed a decrease in large, medium and small LDL fractions such as total lipids, cholesterol, particle concentration or cholesterol esters. Similarly, the MedDiet intervention decreased total cholesterol and cholesterol esters in the large, medium and small VLDL. Literature shows that VLDL concentrations are related directly or indirectly in the development of atherosclerosis [49]; for example the fatty acid composition of VLDL is critical for the activity of lipoprotein lipase and the formation of proatherogenic LDL and VLDL remnants [50]. In the FINRISK cohort, increased risk of cardiovascular disease was associated with all VLDL, IDL, and LDL subclasses, while the L- and M-HDL subclasses were associated with lower risk [51]. Despite the evidence of the role of these metabolites in CVD development, few studies have studied the effect of a MedDiet intervention in different lipids composition of lipoproteins or its subfractions. Interestingly, our results on lipid subfractions agree with a recent publication using the same metabolomic approach, where 47 participants were randomized to a SFA-rich diet, a MUFA-rich diet or a MED diet for 8 weeks. Additionally, in another study, compared to the control group, those participants that replaced SFAs with PUFAs reduced the lipoprotein particle concentration [52]. Finally, olive oil consumption modifies the lipid composition of VLDL [53] as well as the lipoprotein subfractions [54].

In our study, the effect was consistently shown in both groups, usually being stronger in the group undergoing a longer MedDiet intervention/exposure (MedDiet intervention + a self-sustained continuation phase), suggesting that the MedDiet induces favorable changes in metabolites related to CVD disease while the adherence to the MedDiet is sustained. For example, in our study, the ApoB/ApoA1 ratio was decreased in both groups after the intervention and also by adherence to the mMDS, which agrees with other short-term randomized trial with the MedDiet supplemented with olive oil, suggesting that these ratios may predict CVD beyond conventional lipid measures [55]. Finally, we found a significant association with lactate, a metabolite that was previously shown to increase the diabetes risk in the PREDIMED study [56]. However, we found that it occurs in the opposite direction.

### Study Limitations

This was a pilot study with a small sample size. Possibly because of this, most associations lost statistical significance after correcting for multiple testing. However, results were in line with previous research, suggesting that a larger sample size would have retrieved significant results. In addition, the fact that we did not find differences in the mMDS adherence between groups may reflect selection bias since this is a sub-study within 400 firefighters participating in the Feeding America’s Bravest trial, and participants willing to participate could potentially be healthier and more health conscious. Moreover, the control group had higher scores of mMDS at baseline and their scores were slightly improved during the intervention; however, the results were consistent in the cross-sectional analysis. We only adjusted for age and sex, since it was a randomized study with no significant differences between this sub-study and the parent study for the rest of the variables which suggests the need to perform a study that includes larger metabolites. In addition, we did not analyze other metabolites included in other studies nor at baseline for the parent study. In this pilot trial, Group 2 could be considered as the intervention group but Group 1 was not a pure control group because they already finished an active MedDiet intervention and began their self-sustained MedDiet phase. The changes in biomarkers in Group 1 (between time point 1 and 2) are more likely to reflect both residual effects of the MedDiet and continued effects from self-sustained diet intervention. Additionally, most of our participants were male and thus generalizability should be explored. In any case, the results in this study should be corroborated in larger clinical studies with longer follow up due to the pilot study nature and with a powerful study design.

## 5. Conclusions

This MedDiet intervention induces only modest changes in adherence to the MedDiet and consequently in metabolic biomarkers related to lipid metabolism. Further research should confirm these results based on larger study samples in workplace interventions with powerful study designs.

## Figures and Tables

**Table 1:** Characteristics of the firefighters participating in the Feeding America’s Bravest intervention study.

	Time Point 1 (*n* = 48)			Time Point 2 (*n* = 44 *)				
	12 Months MedDiet Intervention (*n* = 24)	Control Group (*n* = 24)	*p*-Value	18 Months MedDiet Intervention (*n* = 22)	Control Group After a 6 Months of Active MedDiet Intervention (*n* = 22)	*p*-Value	*p*-Value (Follow Up-Baseline) Intervention Group	*p*-Value (Follow Up-Baseline) Control Group
Sex, male (%)	91.7	95.8	0.55	84.6	94.1	0.39	N/A	N/A
Age (years)	47.5 (6.7)	47.6 (8.6)	0.95	45.9 (6.7)	49.9 (8.4)	0.17	N/A	N/A
PREDIMED score (0–14 points)	6.1 (2.1)	6.6 (2.1)	0.31	6.4 (1.9)	6.7 (1.9)	0.64	0.48	0.64
mMDs score (0–51 points)	24.2 (6.5)	26.1 (4.9)	0.27	26.0 (6.5)	26.5 (5.6)	0.81	0.23	0.52
Fast food consumption (0–4 points)	2.8 (0.94)	3 (0.61)	0.47	2.76 (0.83)	2.69 (0.63)	0.79	0.06	0.13
Fruit (0–4 points)	1.56 (0.61)	1.65 (0.79)	0.70	1.62 (0.56)	1.71 (0.69)	0.693	0.62	0.71
Vegetable (0–4 points)	1.8 (0.93)	2.11 (0.69)	0.314	1.70 (0.85)	2.06 (0.56)	0.16	0.07	0.11
Sweet desserts (0–4 points)	1 (0.69)	1.24 (0.75)	0.340	0.85 (0.69)	1.35 (0.61)	0.04	0.05	0.84
Cooking oil or fat use at home (0–5 points)	3.0 (2.20)	2.88 (1.99)	0.57	3.85 (1.77)	3.59 (1.87)	0.70	0.07	0.56
Fried food consumption (0–4 points)	0.12 (0.47)	0.35 (0.78)	0.27	0.46 (0.88)	0.71 (0.98)	0.49	0.33	0.33
Breads and starches at home (0–4 points)	2.70 (1.82)	2.39 (1.97)	0.63	1.15 (1.80)	1.41 (1.62)	0.92	0.07	0.21
Ocean fish (0–4 points)	0.78 (0.88)	0.53 (0.72)	0.37	0.39 (0.87)	0.65 (0.86)	0.42	0.09	0.33
Non-alcoholic beverage at home	2.61 (1.72)	3.06 (1.39)	0.41	2.85 (1.41)	3.11 (1.45)	0.61	0.12	0.38
Alcoholic beverages(0–4 points)	1.06 (1.26)	0.94 (1.14)	0.78	1.31 (1.43)	1.12 (1.22)	0.61	1.0	0.58
Wine (0–2 points)	1.58 (0.51)	1.89 (0.31)	0.03	1.61 (0.51)	1.82 (0.39)	0.22	0.35	0.33
Legumes (0–4 points)	3.05 (0.87)	3.06 (0.87)	0.99	3.15 (1.28)	2.53 (1.12)	0.17	0.04	0.04
Nuts (0–4 points)	2.40 (1.09)	2.6 (0.9)	0.57	2.69 (1.11)	2.47 (0.71)	0.61	0.09	0.29

Pairwise comparisons over time include only those participants with complete information at baseline and at the end of follow up (*n* = 23). * At the end of follow up, 44 participants provided information about diet, *n* = 36 plasma serum and diet information, and 30 participants were assessed for anthropometric and other cardiovascular risk factors (Figure S1). Thus, mMDS and PREDIMED included *n* = 44 for the analysis at the end of follow up and for the comparisons.

## Supplementary Materials

The following are available online at https://www.mdpi.com/2072-6643/12/12/3610/s1, Figure S1: Flowchart of participants at time point 1 and 2 of the Feeding America’s Bravest trial, Figure S2: Associations of unit changes in mMDS score by SD changes in plasma biomarkers between month 6 (time point 2) and baseline (time point 1) in all participants grouped together, Figure S3: Association between biomarkers and the PREDIMED score adherence (cross-sectional analysis), Table S1: The 6 months effect of the Mediterranean Diet intervention on metabolic biomarkers (linear mixed model analysis with *p*-values corrected for multiple testing).
