# Supplementary material for: The Effects of a Mediterranean Diet Intervention on Targeted Plasma Metabolic Biomarkers among US Firefighters: A Pilot Cluster-Randomized Trial
Source: Nutrients. 2020 Nov 24;12(12):3610. doi: 10.3390/nu12123610 (PMC7761450; doi:10.3390/nu12123610)
Supplement: Supplementary file 1 [file nutrients-12-03610-s001.pdf]

ONLINE SUPPLEMENTARY INFORMATION

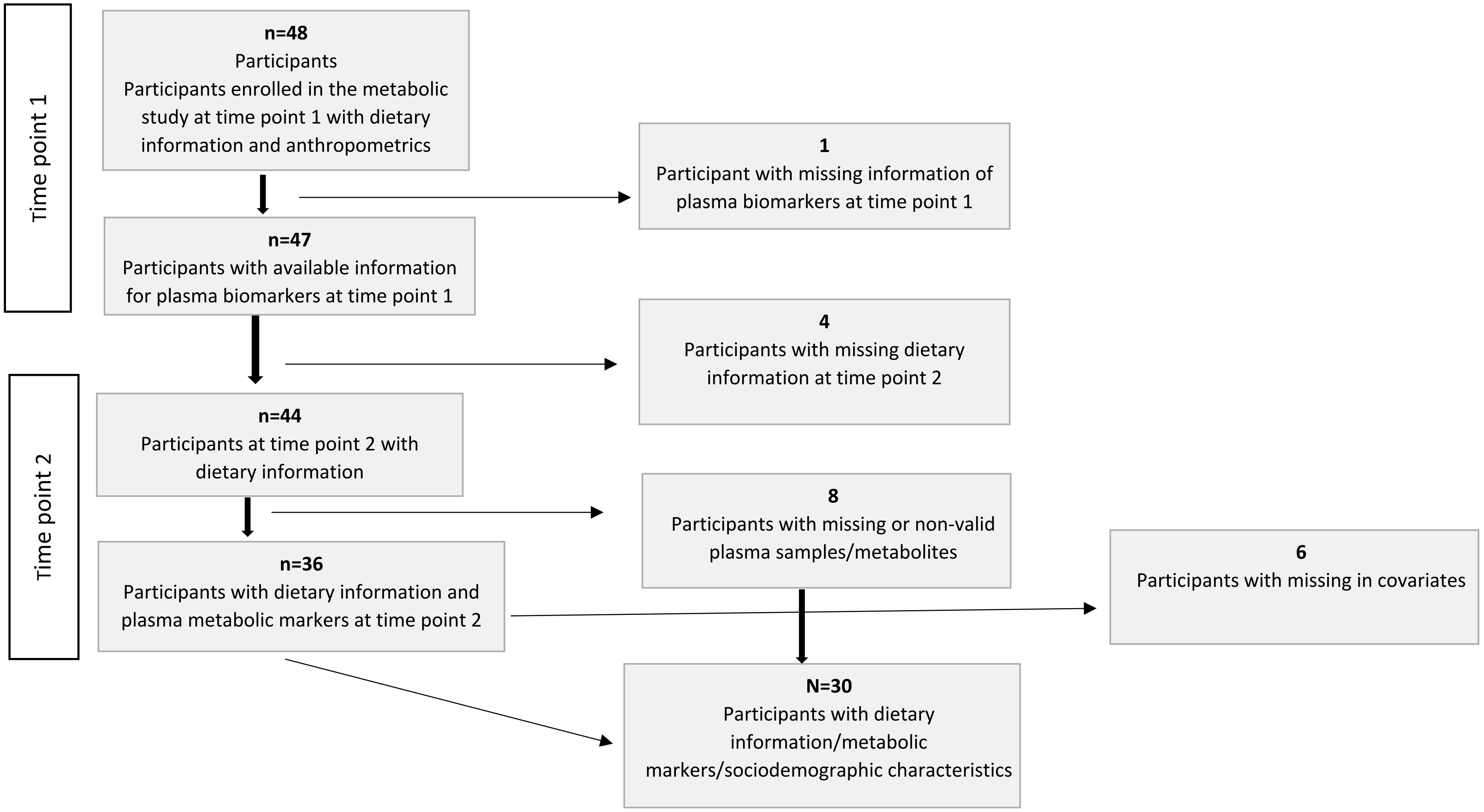

Figure S1. Flowchart of participants at time point 1 and 2 of the Feeding America's Bravest trial.

Figure S2. Associations of unit changes in mMDS score by SD changes in plasma biomarkers between month 6 (time point 2) and baseline (time point 1) in all participants grouped together.

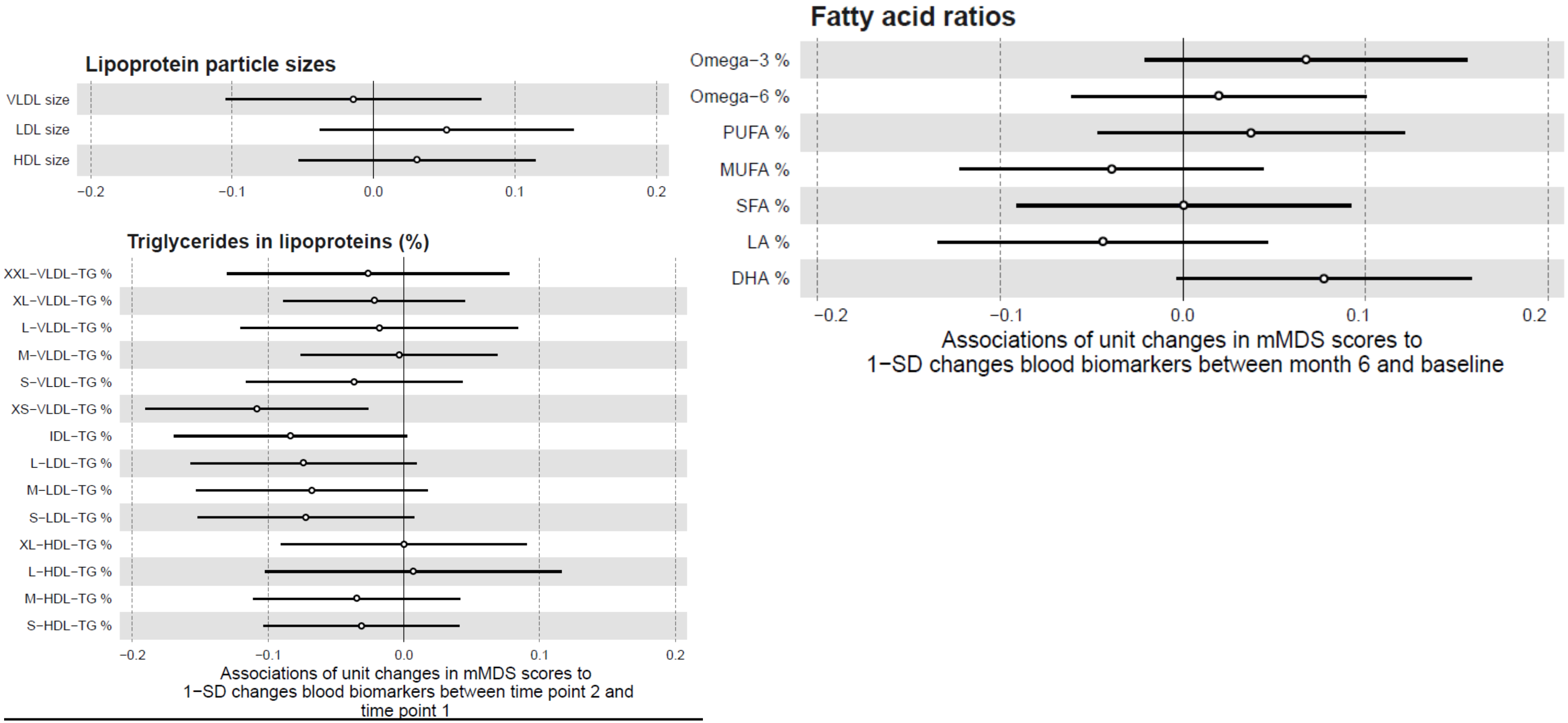

Figure S3. Association between biomarkers and the PREDIMED Score adherence (cross-sectional analysis). Red lines show the results for the participants at Baseline and black lines for the participants at follow up). Results show changes by SD and are displayed by hollow points and significant results by filled points along with their 95% confidence intervals

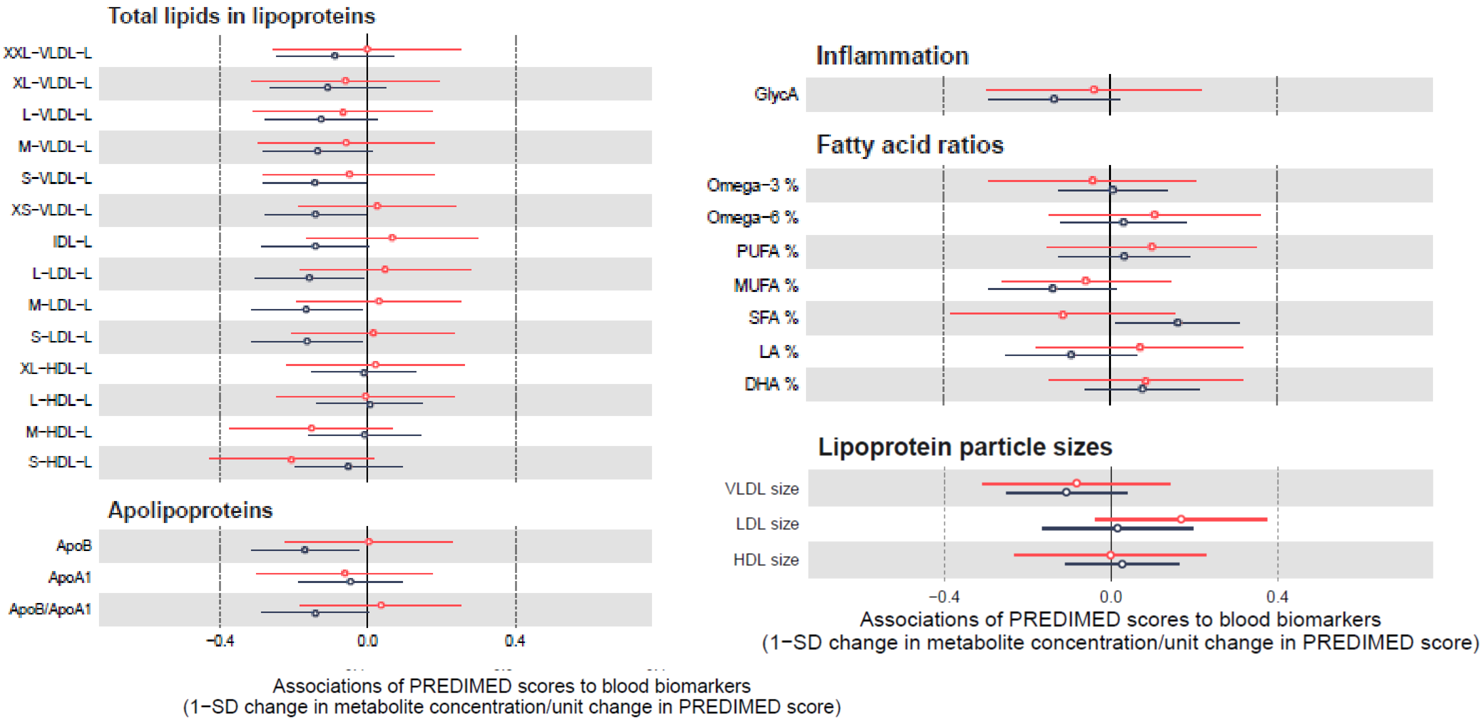

Table S1. Six-month effect of the Mediterranean Diet intervention on metabolic biomarkers (linear mixed model analysis with p-values corrected for multiple testing)

Group	Biomarker	Estimate (per 1-SD change in biomarkers)	SE	P-Value
Control	Total_C	-0.192	0.176	0.295
Intervention	Total_C	-0.330	0.189	0.104
Control	VLDL_C	-0.132	0.177	0.469
Intervention	VLDL_C	-0.337	0.186	0.094
Control	Remnant_C	-0.247	0.188	0.212
Intervention	Remnant_C	-0.410	0.209	0.072
Control	LDL_C	-0.328	0.207	0.137
Intervention	LDL_C	-0.439	0.197	0.045
Control	HDL_C	0.215	0.094	0.040
Intervention	HDL_C	0.146	0.095	0.148
Control	HDL2_C	0.210	0.087	0.032
Intervention	HDL2_C	0.165	0.091	0.094
Control	HDL3_C	0.130	0.234	0.589
Intervention	HDL3_C	-0.123	0.142	0.403
Control	Total_CE	-0.202	0.174	0.267
Intervention	Total_CE	-0.343	0.185	0.086
Control	Total_FC	-0.148	0.191	0.452
Intervention	Total_FC	-0.315	0.198	0.135
Control	Total_TG	0.106	0.151	0.495
Intervention	Total_TG	-0.087	0.134	0.529
Control	VLDL_TG	0.096	0.156	0.548
Intervention	VLDL_TG	-0.090	0.140	0.530
Control	LDL_TG	0.144	0.206	0.497
Intervention	LDL_TG	-0.077	0.153	0.622
Control	HDL_TG	0.148	0.190	0.452
Intervention	HDL_TG	-0.125	0.171	0.477
Control	Phosphoglyc	0.264	0.171	0.146
Intervention	Phosphoglyc	-0.026	0.161	0.874
Control	TG_by_PG	0.008	0.167	0.964
Intervention	TG_by_PG	-0.078	0.127	0.550
Control	Cholines	0.274	0.178	0.148
Intervention	Cholines	-0.066	0.170	0.705
Control	Phosphatidylc	0.206	0.192	0.301
Intervention	Phosphatidylc	-0.032	0.168	0.852
Control	Sphingomyelins	-0.203	0.304	0.514
Intervention	Sphingomyelins	0.006	0.219	0.979
Control	ApoB	-0.177	0.176	0.334
Intervention	ApoB	-0.367	0.187	0.071
Control	ApoA1	0.177	0.104	0.112
Intervention	ApoA1	0.028	0.127	0.827
Control	ApoB_by_ApoA1	-0.276	0.145	0.078
Intervention	ApoB_by_ApoA1	-0.354	0.145	0.030

Control	Total_FA	0.070	0.168	0.683
Intervention	Total_FA	-0.167	0.150	0.286
Control	Unsaturation	-0.372	0.126	0.011
Intervention	Unsaturation	-0.135	0.184	0.476
Control	Omega_3	0.010	0.112	0.928
Intervention	Omega_3	-0.081	0.202	0.697
Control	Omega_6	0.018	0.158	0.913
Intervention	Omega_6	-0.283	0.167	0.114
Control	PUFA	0.015	0.153	0.924
Intervention	PUFA	-0.244	0.163	0.157
Control	MUFA	0.076	0.196	0.705
Intervention	MUFA	-0.105	0.156	0.513
Control	SFA	0.121	0.160	0.462
Intervention	SFA	-0.158	0.155	0.325
Control	LA	0.094	0.187	0.625
Intervention	LA	-0.228	0.183	0.234
Control	DHA	-0.022	0.190	0.911
Intervention	DHA	0.102	0.228	0.663
Control	Omega_3_pct	-0.144	0.142	0.327
Intervention	Omega_3_pct	0.212	0.303	0.495
Control	Omega_6_pct	-0.167	0.186	0.386
Intervention	Omega_6_pct	-0.203	0.187	0.297
Control	PUFA_pct	-0.198	0.206	0.353
Intervention	PUFA_pct	-0.149	0.209	0.488
Control	MUFA_pct	0.096	0.296	0.751
Intervention	MUFA_pct	0.173	0.184	0.363
Control	SFA_pct	0.339	0.294	0.269
Intervention	SFA_pct	0.018	0.336	0.959
Control	LA_pct	0.051	0.258	0.846
Intervention	LA_pct	-0.139	0.274	0.620
Control	DHA_pct	-0.239	0.370	0.529
Intervention	DHA_pct	0.463	0.295	0.140
Control	Ala	0.569	0.266	0.052
Intervention	Ala	0.081	0.159	0.618
Control	Gln	-0.109	0.255	0.676
Intervention	Gln	-0.186	0.217	0.407
Control	His	-0.271	0.350	0.452
Intervention	His	-0.964	0.342	0.015
Control	Ile	0.240	0.285	0.414
Intervention	Ile	0.148	0.357	0.685
Control	Leu	0.332	0.310	0.304
Intervention	Leu	-0.066	0.378	0.865
Control	Val	0.308	0.269	0.272
Intervention	Val	0.383	0.364	0.313
Control	Phe	0.443	0.312	0.180
Intervention	Phe	-0.264	0.370	0.489

Control	Tyr	0.558	0.348	0.133
Intervention	Tyr	-0.154	0.290	0.604
Control	Glucose	0.268	0.103	0.022
Intervention	Glucose	-0.268	0.188	0.177
Control	Lactate	-0.056	0.386	0.886
Intervention	Lactate	0.815	0.200	0.001
Control	Citrate	-0.184	0.296	0.545
Intervention	Citrate	-0.139	0.265	0.610
Control	Acetate	-0.050	0.317	0.878
Intervention	Acetate	0.125	0.213	0.567
Control	Acetoacetate	-0.245	0.160	0.150
Intervention	Acetoacetate	-0.006	0.296	0.985
Control	bOHbutyrate	-0.378	0.185	0.065
Intervention	bOHbutyrate	0.072	0.372	0.850
Control	Creatinine	0.485	0.208	0.036
Intervention	Creatinine	0.009	0.139	0.952
Control	Albumin	-0.022	0.400	0.957
Intervention	Albumin	0.099	0.333	0.770
Control	GlycA	-0.166	0.191	0.399
Intervention	GlycA	-0.135	0.119	0.278
Control	XXL_VLDL_P	-0.013	0.228	0.954
Intervention	XXL_VLDL_P	-0.336	0.202	0.120
Control	XXL_VLDL_L	-0.013	0.232	0.955
Intervention	XXL_VLDL_L	-0.344	0.205	0.117
Control	XXL_VLDL_PL	0.009	0.221	0.968
Intervention	XXL_VLDL_PL	-0.302	0.189	0.133
Control	XXL_VLDL_C	-0.086	0.224	0.707
Intervention	XXL_VLDL_C	-0.306	0.190	0.131
Control	XXL_VLDL_CE	-0.140	0.225	0.545
Intervention	XXL_VLDL_CE	-0.329	0.191	0.108
Control	XXL_VLDL_FC	-0.012	0.222	0.957
Intervention	XXL_VLDL_FC	-0.267	0.190	0.184
Control	XXL_VLDL_TG	-0.002	0.233	0.994
Intervention	XXL_VLDL_TG	-0.352	0.209	0.117
Control	XL_VLDL_P	0.063	0.175	0.726
Intervention	XL_VLDL_P	-0.146	0.153	0.359
Control	XL_VLDL_L	0.061	0.187	0.748
Intervention	XL_VLDL_L	-0.169	0.161	0.314
Control	XL_VLDL_PL	0.022	0.188	0.908
Intervention	XL_VLDL_PL	-0.190	0.163	0.265
Control	XL_VLDL_C	-0.048	0.210	0.824
Intervention	XL_VLDL_C	-0.242	0.173	0.186
Control	XL_VLDL_CE	-0.031	0.198	0.880
Intervention	XL_VLDL_CE	-0.220	0.164	0.204
Control	XL_VLDL_FC	-0.069	0.221	0.760
Intervention	XL_VLDL_FC	-0.263	0.183	0.174

Control	XL_VLDL_TG	0.096	0.174	0.591
Intervention	XL_VLDL_TG	-0.131	0.153	0.408
Control	L_VLDL_P	0.069	0.151	0.654
Intervention	L_VLDL_P	-0.081	0.143	0.581
Control	L_VLDL_L	0.091	0.167	0.597
Intervention	L_VLDL_L	-0.098	0.155	0.538
Control	L_VLDL_PL	0.068	0.153	0.666
Intervention	L_VLDL_PL	-0.099	0.147	0.510
Control	L_VLDL_C	0.040	0.161	0.808
Intervention	L_VLDL_C	-0.134	0.152	0.393
Control	L_VLDL_CE	0.023	0.151	0.880
Intervention	L_VLDL_CE	-0.122	0.148	0.422
Control	L_VLDL_FC	0.054	0.167	0.749
Intervention	L_VLDL_FC	-0.139	0.154	0.384
Control	L_VLDL_TG	0.097	0.162	0.562
Intervention	L_VLDL_TG	-0.076	0.151	0.625
Control	M_VLDL_P	0.040	0.143	0.787
Intervention	M_VLDL_P	-0.110	0.138	0.440
Control	M_VLDL_L	0.051	0.160	0.752
Intervention	M_VLDL_L	-0.125	0.147	0.412
Control	M_VLDL_PL	0.040	0.148	0.789
Intervention	M_VLDL_PL	-0.122	0.142	0.407
Control	M_VLDL_C	-0.013	0.158	0.936
Intervention	M_VLDL_C	-0.184	0.156	0.259
Control	M_VLDL_CE	-0.080	0.157	0.620
Intervention	M_VLDL_CE	-0.239	0.165	0.171
Control	M_VLDL_FC	0.060	0.151	0.697
Intervention	M_VLDL_FC	-0.114	0.143	0.442
Control	M_VLDL_TG	0.073	0.155	0.644
Intervention	M_VLDL_TG	-0.091	0.141	0.532
Control	S_VLDL_P	0.044	0.141	0.761
Intervention	S_VLDL_P	-0.140	0.152	0.374
Control	S_VLDL_L	0.037	0.154	0.812
Intervention	S_VLDL_L	-0.157	0.154	0.327
Control	S_VLDL_PL	0.100	0.154	0.526
Intervention	S_VLDL_PL	-0.114	0.155	0.477
Control	S_VLDL_C	-0.134	0.179	0.467
Intervention	S_VLDL_C	-0.348	0.193	0.095
Control	S_VLDL_CE	-0.277	0.203	0.196
Intervention	S_VLDL_CE	-0.471	0.217	0.049
Control	S_VLDL_FC	0.075	0.157	0.639
Intervention	S_VLDL_FC	-0.151	0.159	0.362
Control	S_VLDL_TG	0.107	0.141	0.462
Intervention	S_VLDL_TG	-0.053	0.133	0.700
Control	XS_VLDL_P	-0.185	0.163	0.278
Intervention	XS_VLDL_P	-0.364	0.189	0.076

Control	XS_VLDL_L	-0.237	0.167	0.180
Intervention	XS_VLDL_L	-0.390	0.197	0.069
Control	XS_VLDL_PL	-0.205	0.175	0.263
Intervention	XS_VLDL_PL	-0.338	0.180	0.083
Control	XS_VLDL_C	-0.422	0.229	0.088
Intervention	XS_VLDL_C	-0.559	0.246	0.041
Control	XS_VLDL_CE	-0.457	0.265	0.108
Intervention	XS_VLDL_CE	-0.574	0.267	0.051
Control	XS_VLDL_FC	-0.251	0.188	0.204
Intervention	XS_VLDL_FC	-0.438	0.195	0.043
Control	XS_VLDL_TG	0.136	0.167	0.429
Intervention	XS_VLDL_TG	-0.014	0.140	0.922
Control	IDL_P	-0.273	0.188	0.170
Intervention	IDL_P	-0.418	0.204	0.061
Control	IDL_L	-0.313	0.185	0.115
Intervention	IDL_L	-0.403	0.206	0.072
Control	IDL_PL	-0.309	0.192	0.132
Intervention	IDL_PL	-0.435	0.194	0.043
Control	IDL_C	-0.355	0.202	0.102
Intervention	IDL_C	-0.440	0.218	0.065
Control	IDL_CE	-0.362	0.212	0.112
Intervention	IDL_CE	-0.448	0.231	0.075
Control	IDL_FC	-0.296	0.186	0.136
Intervention	IDL_FC	-0.424	0.181	0.036
Control	IDL_TG	0.156	0.216	0.484
Intervention	IDL_TG	0.001	0.152	0.995
Control	L_LDL_P	-0.257	0.199	0.219
Intervention	L_LDL_P	-0.427	0.201	0.054
Control	L_LDL_L	-0.297	0.192	0.147
Intervention	L_LDL_L	-0.404	0.200	0.064
Control	L_LDL_PL	-0.276	0.195	0.181
Intervention	L_LDL_PL	-0.400	0.207	0.075
Control	L_LDL_C	-0.326	0.202	0.130
Intervention	L_LDL_C	-0.439	0.203	0.050
Control	L_LDL_CE	-0.318	0.204	0.143
Intervention	L_LDL_CE	-0.447	0.205	0.048
Control	L_LDL_FC	-0.311	0.199	0.141
Intervention	L_LDL_FC	-0.426	0.197	0.050
Control	L_LDL_TG	0.119	0.209	0.578
Intervention	L_LDL_TG	-0.078	0.160	0.633
Control	M_LDL_P	-0.233	0.213	0.295
Intervention	M_LDL_P	-0.438	0.200	0.047
Control	M_LDL_L	-0.268	0.209	0.223
Intervention	M_LDL_L	-0.423	0.198	0.052
Control	M_LDL_PL	-0.214	0.197	0.296
Intervention	M_LDL_PL	-0.379	0.213	0.099

Control	M_LDL_C	-0.304	0.221	0.191
Intervention	M_LDL_C	-0.463	0.199	0.037
Control	M_LDL_CE	-0.301	0.221	0.196
Intervention	M_LDL_CE	-0.468	0.196	0.033
Control	M_LDL_FC	-0.288	0.224	0.221
Intervention	M_LDL_FC	-0.462	0.219	0.055
Control	M_LDL_TG	0.187	0.239	0.448
Intervention	M_LDL_TG	-0.051	0.168	0.765
Control	S_LDL_P	-0.195	0.213	0.377
Intervention	S_LDL_P	-0.434	0.199	0.048
Control	S_LDL_L	-0.225	0.213	0.310
Intervention	S_LDL_L	-0.430	0.198	0.049
Control	S_LDL_PL	-0.131	0.203	0.531
Intervention	S_LDL_PL	-0.361	0.214	0.115
Control	S_LDL_C	-0.280	0.225	0.235
Intervention	S_LDL_C	-0.472	0.198	0.033
Control	S_LDL_CE	-0.280	0.221	0.227
Intervention	S_LDL_CE	-0.463	0.194	0.033
Control	S_LDL_FC	-0.255	0.246	0.319
Intervention	S_LDL_FC	-0.515	0.221	0.037
Control	S_LDL_TG	0.142	0.163	0.399
Intervention	S_LDL_TG	-0.116	0.139	0.418
Control	XL_HDL_P	0.074	0.121	0.554
Intervention	XL_HDL_P	-0.038	0.147	0.800
Control	XL_HDL_L	0.080	0.142	0.584
Intervention	XL_HDL_L	-0.057	0.163	0.732
Control	XL_HDL_PL	0.156	0.149	0.312
Intervention	XL_HDL_PL	0.096	0.161	0.562
Control	XL_HDL_C	-0.010	0.155	0.949
Intervention	XL_HDL_C	-0.194	0.169	0.273
Control	XL_HDL_CE	-0.025	0.172	0.886
Intervention	XL_HDL_CE	-0.250	0.179	0.186
Control	XL_HDL_FC	0.026	0.118	0.825
Intervention	XL_HDL_FC	-0.036	0.142	0.802
Control	XL_HDL_TG	-0.121	0.287	0.680
Intervention	XL_HDL_TG	-0.492	0.277	0.100
Control	L_HDL_P	0.171	0.086	0.069
Intervention	L_HDL_P	0.115	0.086	0.205
Control	L_HDL_L	0.177	0.089	0.067
Intervention	L_HDL_L	0.102	0.078	0.217
Control	L_HDL_PL	0.185	0.082	0.042
Intervention	L_HDL_PL	0.113	0.079	0.178
Control	L_HDL_C	0.163	0.102	0.134
Intervention	L_HDL_C	0.121	0.090	0.200
Control	L_HDL_CE	0.167	0.101	0.123
Intervention	L_HDL_CE	0.122	0.090	0.197

Control	L_HDL_FC	0.137	0.104	0.208
Intervention	L_HDL_FC	0.130	0.092	0.180
Control	L_HDL_TG	0.140	0.148	0.363
Intervention	L_HDL_TG	-0.153	0.149	0.324
Control	M_HDL_P	0.498	0.147	0.005
Intervention	M_HDL_P	0.386	0.195	0.069
Control	M_HDL_L	0.485	0.146	0.006
Intervention	M_HDL_L	0.387	0.181	0.052
Control	M_HDL_PL	0.524	0.158	0.006
Intervention	M_HDL_PL	0.353	0.194	0.091
Control	M_HDL_C	0.412	0.113	0.003
Intervention	M_HDL_C	0.390	0.159	0.029
Control	M_HDL_CE	0.400	0.111	0.003
Intervention	M_HDL_CE	0.403	0.158	0.024
Control	M_HDL_FC	0.455	0.129	0.004
Intervention	M_HDL_FC	0.336	0.171	0.072
Control	M_HDL_TG	0.187	0.178	0.312
Intervention	M_HDL_TG	0.054	0.167	0.751
Control	S_HDL_P	0.518	0.223	0.037
Intervention	S_HDL_P	0.313	0.263	0.255
Control	S_HDL_L	0.505	0.227	0.044
Intervention	S_HDL_L	0.319	0.253	0.230
Control	S_HDL_PL	0.593	0.241	0.029
Intervention	S_HDL_PL	0.401	0.286	0.184
Control	S_HDL_C	0.248	0.320	0.452
Intervention	S_HDL_C	0.177	0.262	0.512
Control	S_HDL_CE	0.178	0.332	0.601
Intervention	S_HDL_CE	0.138	0.264	0.610
Control	S_HDL_FC	0.600	0.190	0.008
Intervention	S_HDL_FC	0.353	0.292	0.248
Control	S_HDL_TG	0.135	0.141	0.358
Intervention	S_HDL_TG	-0.050	0.117	0.676
Control	VLDL_size	0.218	0.187	0.265
Intervention	VLDL_size	-0.005	0.151	0.972
Control	LDL_size	-0.262	0.249	0.311
Intervention	LDL_size	0.036	0.232	0.880
Control	HDL_size	0.064	0.104	0.546
Intervention	HDL_size	0.032	0.095	0.738
Control	XXL_VLDL_PL_pct	0.601	0.407	0.168
Intervention	XXL_VLDL_PL_pct	0.005	0.176	0.978
Control	XXL_VLDL_C_pct	-0.223	0.348	0.534
Intervention	XXL_VLDL_C_pct	-0.189	0.186	0.349
Control	XXL_VLDL_CE_pct	-0.692	0.368	0.087
Intervention	XXL_VLDL_CE_pct	-0.152	0.172	0.409
Control	XXL_VLDL_FC_pct	0.428	0.301	0.183
Intervention	XXL_VLDL_FC_pct	-0.193	0.207	0.386

Control	XXL_VLDL_TG_pct	0.021	0.148	0.891
Intervention	XXL_VLDL_TG_pct	0.192	0.194	0.359
Control	XL_VLDL_PL_pct	0.397	0.328	0.257
Intervention	XL_VLDL_PL_pct	-0.183	0.061	0.015
Control	XL_VLDL_C_pct	0.004	0.329	0.991
Intervention	XL_VLDL_C_pct	-0.273	0.158	0.117
Control	XL_VLDL_CE_pct	0.058	0.336	0.868
Intervention	XL_VLDL_CE_pct	-0.151	0.148	0.335
Control	XL_VLDL_FC_pct	-0.093	0.385	0.815
Intervention	XL_VLDL_FC_pct	-0.498	0.228	0.056
Control	XL_VLDL_TG_pct	0.034	0.197	0.866
Intervention	XL_VLDL_TG_pct	0.302	0.125	0.039
Control	L_VLDL_PL_pct	-0.003	0.030	0.909
Intervention	L_VLDL_PL_pct	-0.034	0.029	0.260
Control	L_VLDL_C_pct	-0.125	0.067	0.087
Intervention	L_VLDL_C_pct	-0.303	0.116	0.026
Control	L_VLDL_CE_pct	-0.265	0.068	0.002
Intervention	L_VLDL_CE_pct	-0.064	0.067	0.365
Control	L_VLDL_FC_pct	0.129	0.142	0.383
Intervention	L_VLDL_FC_pct	-0.692	0.374	0.094
Control	L_VLDL_TG_pct	0.176	0.101	0.110
Intervention	L_VLDL_TG_pct	0.395	0.145	0.021
Control	M_VLDL_PL_pct	-0.114	0.254	0.661
Intervention	M_VLDL_PL_pct	-0.049	0.239	0.839
Control	M_VLDL_C_pct	-0.252	0.096	0.020
Intervention	M_VLDL_C_pct	-0.190	0.125	0.153
Control	M_VLDL_CE_pct	-0.417	0.079	0.010
Intervention	M_VLDL_CE_pct	-0.349	0.143	0.030
Control	M_VLDL_FC_pct	0.109	0.059	0.089
Intervention	M_VLDL_FC_pct	0.145	0.188	0.455
Control	M_VLDL_TG_pct	0.296	0.107	0.016
Intervention	M_VLDL_TG_pct	0.231	0.127	0.091
Control	S_VLDL_PL_pct	0.283	0.191	0.162
Intervention	S_VLDL_PL_pct	0.318	0.211	0.155
Control	S_VLDL_C_pct	-0.515	0.177	0.012
Intervention	S_VLDL_C_pct	-0.512	0.161	0.007
Control	S_VLDL_CE_pct	-0.595	0.180	0.006
Intervention	S_VLDL_CE_pct	-0.538	0.176	0.009
Control	S_VLDL_FC_pct	0.308	0.285	0.300
Intervention	S_VLDL_FC_pct	0.064	0.132	0.637
Control	S_VLDL_TG_pct	0.317	0.151	0.056
Intervention	S_VLDL_TG_pct	0.329	0.110	0.010
Control	XS_VLDL_PL_pct	0.066	0.235	0.783
Intervention	XS_VLDL_PL_pct	0.186	0.216	0.404
Control	XS_VLDL_C_pct	-0.588	0.335	0.103
Intervention	XS_VLDL_C_pct	-0.444	0.251	0.100

Control	XS_VLDL_CE_pct	-0.578	0.347	0.120
Intervention	XS_VLDL_CE_pct	-0.407	0.286	0.177
Control	XS_VLDL_FC_pct	-0.121	0.283	0.676
Intervention	XS_VLDL_FC_pct	-0.156	0.167	0.370
Control	XS_VLDL_TG_pct	0.459	0.224	0.061
Intervention	XS_VLDL_TG_pct	0.360	0.180	0.067
Control	IDL_PL_pct	0.196	0.172	0.275
Intervention	IDL_PL_pct	0.071	0.240	0.773
Control	IDL_C_pct	-0.547	0.286	0.078
Intervention	IDL_C_pct	-0.380	0.275	0.191
Control	IDL_CE_pct	-0.505	0.309	0.126
Intervention	IDL_CE_pct	-0.304	0.327	0.369
Control	IDL_FC_pct	-0.114	0.184	0.544
Intervention	IDL_FC_pct	-0.164	0.123	0.207
Control	IDL_TG_pct	0.514	0.279	0.089
Intervention	IDL_TG_pct	0.428	0.195	0.047
Control	L_LDL_PL_pct	0.347	0.187	0.086
Intervention	L_LDL_PL_pct	0.349	0.171	0.062
Control	L_LDL_C_pct	-0.563	0.247	0.040
Intervention	L_LDL_C_pct	-0.491	0.204	0.032
Control	L_LDL_CE_pct	-0.443	0.208	0.053
Intervention	L_LDL_CE_pct	-0.399	0.217	0.089
Control	L_LDL_FC_pct	-0.041	0.141	0.777
Intervention	L_LDL_FC_pct	0.029	0.137	0.837
Control	L_LDL_TG_pct	0.541	0.296	0.091
Intervention	L_LDL_TG_pct	0.456	0.192	0.033
Control	M_LDL_PL_pct	0.373	0.261	0.177
Intervention	M_LDL_PL_pct	0.386	0.141	0.017
Control	M_LDL_C_pct	-0.492	0.230	0.052
Intervention	M_LDL_C_pct	-0.447	0.135	0.006
Control	M_LDL_CE_pct	-0.414	0.193	0.051
Intervention	M_LDL_CE_pct	-0.364	0.129	0.014
Control	M_LDL_FC_pct	0.190	0.150	0.229
Intervention	M_LDL_FC_pct	0.220	0.140	0.141
Control	M_LDL_TG_pct	0.610	0.285	0.052
Intervention	M_LDL_TG_pct	0.509	0.211	0.031
Control	S_LDL_PL_pct	0.430	0.239	0.095
Intervention	S_LDL_PL_pct	0.428	0.148	0.013
Control	S_LDL_C_pct	-0.487	0.231	0.055
Intervention	S_LDL_C_pct	-0.397	0.130	0.009
Control	S_LDL_CE_pct	-0.415	0.194	0.052
Intervention	S_LDL_CE_pct	-0.308	0.123	0.026
Control	S_LDL_FC_pct	0.096	0.134	0.484
Intervention	S_LDL_FC_pct	0.027	0.140	0.849
Control	S_LDL_TG_pct	0.439	0.210	0.056
Intervention	S_LDL_TG_pct	0.298	0.126	0.034

Control	XL_HDL_PL_pct	0.205	0.176	0.274
Intervention	XL_HDL_PL_pct	0.347	0.146	0.044
Control	XL_HDL_C_pct	-0.256	0.266	0.361
Intervention	XL_HDL_C_pct	-0.606	0.350	0.122
Control	XL_HDL_CE_pct	-0.251	0.296	0.418
Intervention	XL_HDL_CE_pct	-0.704	0.313	0.054
Control	XL_HDL_FC_pct	-0.189	0.130	0.182
Intervention	XL_HDL_FC_pct	-0.079	0.416	0.854
Control	XL_HDL_TG_pct	-0.361	0.330	0.302
Intervention	XL_HDL_TG_pct	-0.568	0.436	0.228
Control	L_HDL_PL_pct	-0.137	0.164	0.422
Intervention	L_HDL_PL_pct	0.070	0.185	0.713
Control	L_HDL_C_pct	0.151	0.164	0.379
Intervention	L_HDL_C_pct	-0.083	0.291	0.781
Control	L_HDL_CE_pct	0.157	0.203	0.455
Intervention	L_HDL_CE_pct	-0.059	0.282	0.838
Control	L_HDL_FC_pct	0.123	0.110	0.285
Intervention	L_HDL_FC_pct	-0.192	0.341	0.585
Control	L_HDL_TG_pct	-0.098	0.288	0.739
Intervention	L_HDL_TG_pct	-0.368	0.297	0.241
Control	M_HDL_PL_pct	-0.005	0.152	0.977
Intervention	M_HDL_PL_pct	-0.384	0.153	0.026
Control	M_HDL_C_pct	0.045	0.107	0.680
Intervention	M_HDL_C_pct	0.248	0.135	0.089
Control	M_HDL_CE_pct	-0.034	0.141	0.813
Intervention	M_HDL_CE_pct	0.242	0.149	0.128
Control	M_HDL_FC_pct	0.253	0.140	0.094
Intervention	M_HDL_FC_pct	0.212	0.106	0.066
Control	M_HDL_TG_pct	-0.074	0.135	0.595
Intervention	M_HDL_TG_pct	-0.112	0.113	0.340
Control	S_HDL_PL_pct	0.118	0.395	0.770
Intervention	S_HDL_PL_pct	0.130	0.296	0.668
Control	S_HDL_C_pct	-0.122	0.330	0.718
Intervention	S_HDL_C_pct	-0.015	0.249	0.952
Control	S_HDL_CE_pct	-0.134	0.323	0.684
Intervention	S_HDL_CE_pct	-0.013	0.245	0.957
Control	S_HDL_FC_pct	0.196	0.274	0.487
Intervention	S_HDL_FC_pct	0.103	0.266	0.705
Control	S_HDL_TG_pct	0.001	0.109	0.990
Intervention	S_HDL_TG_pct	-0.134	0.077	0.105
